# Supplementary material for: Substrate-binding destabilizes the hydrophobic cluster to relieve the autoinhibition of bacterial ubiquitin ligase IpaH9.8
Source: Commun Biol. 2020 Dec 10;3:752. doi: 10.1038/s42003-020-01492-1 (PMC7728815; doi:10.1038/s42003-020-01492-1)
Supplement: Supplementary file 2 — Supplementary Information [file 42003_2020_1492_MOESM2_ESM.pdf]

Supplementary Information

Substrate-binding destabilizes the hydrophobic cluster to relieve the autoinhibition of bacterial ubiquitin ligase IpaH9.8

Yuxin Ye[1,2 #]*, Yuxian Xiong[1,3 #], and Hao Huang[1,3] *

[1]State Key Laboratory of Chemical Oncogenomics, School of Chemical Biology and Biotechnology, Peking University Shenzhen Graduate School, Shenzhen, 518055, China; [2]Shenzhen Bay Laboratory Pingshan Translational Medicine Center, Shenzhen, China; [3]Laboratory of Structural Biology and Drug Discovery, Peking University Shenzhen Graduate School, Shenzhen, 518055, China;

[#]These authors contributed equally.

*Co-corresponding authors.

Dr. Yuxin Ye, Tel: +86-0755-2603-2321; E-mail: yeyx@pkusz.edu.cn

Dr. Hao Huang, Tel: +86-0755-2603-2321; E-mail: huang.hao@pku.edu.cn

Supplementary Figures

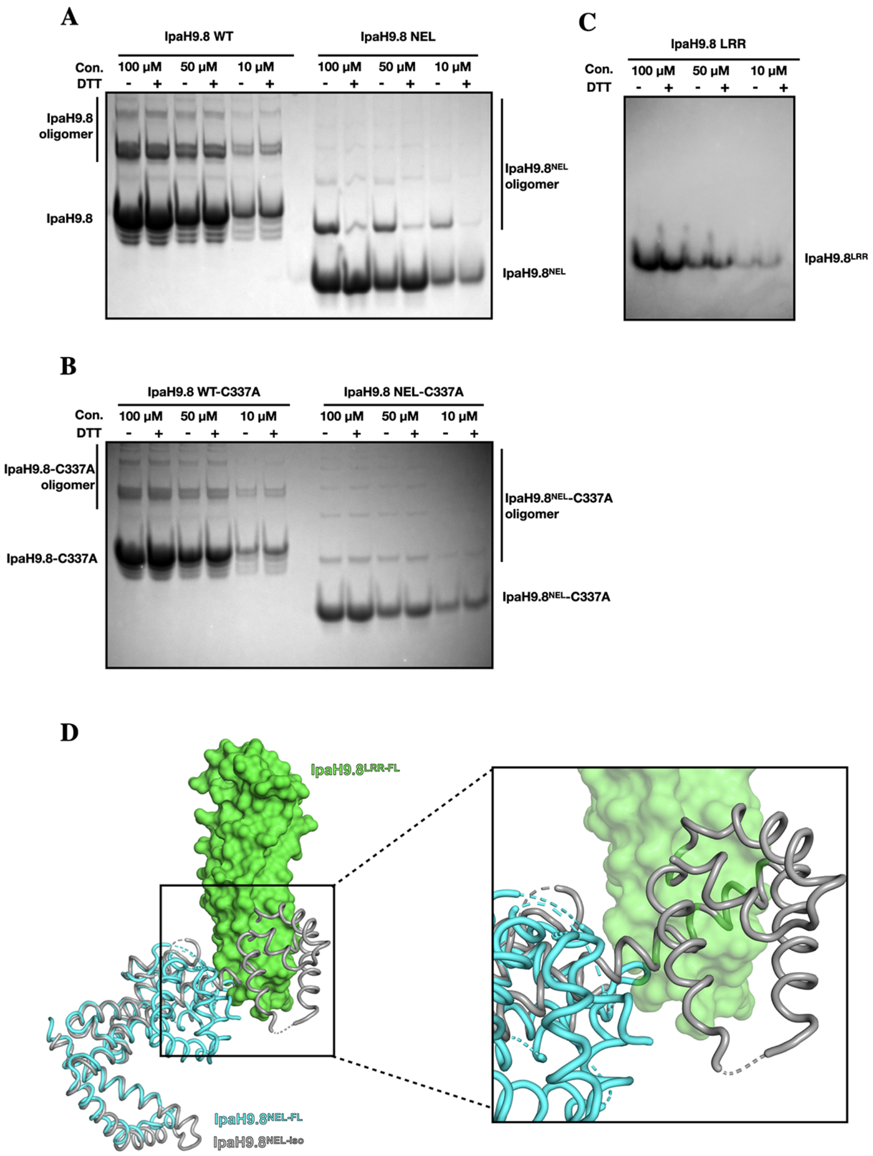

Supplementary Figure 1. ***The Native-PAGE of IpaH9.8 and the structure superposition of IpaH9.8$^{NEL\text{-}iso}$ and IpaH9.8***

(A). The Native-PAGE of wild-type IpaH9.8 and IpaH9.8^{NEL} at different concentrations (100 μM, 50 μM, 10 μM), under reducing (5 mM DTT) and non-reducing conditions. (B). The Native-PAGE of IpaH9.8-C337A and IpaH9.8^{NEL}-C337A in similar conditions to (A). (C). The Native-PAGE of IpaH9.8^{LRR} in similar conditions to (A) and (B). (D). *Left*, superposition of IpaH9.8 (LRR colored in green, and NEL colored in cyan) and IpaH9.8$^{NEL\text{-}iso}$ (PDBID: 3L3P, colored in gray) structures. *Right*, zoomed-in view of the N-subdomain of NEL in IpaH9.8$^{NEL\text{-}iso}$, which overlaps with the LRR domain of IpaH9.8.

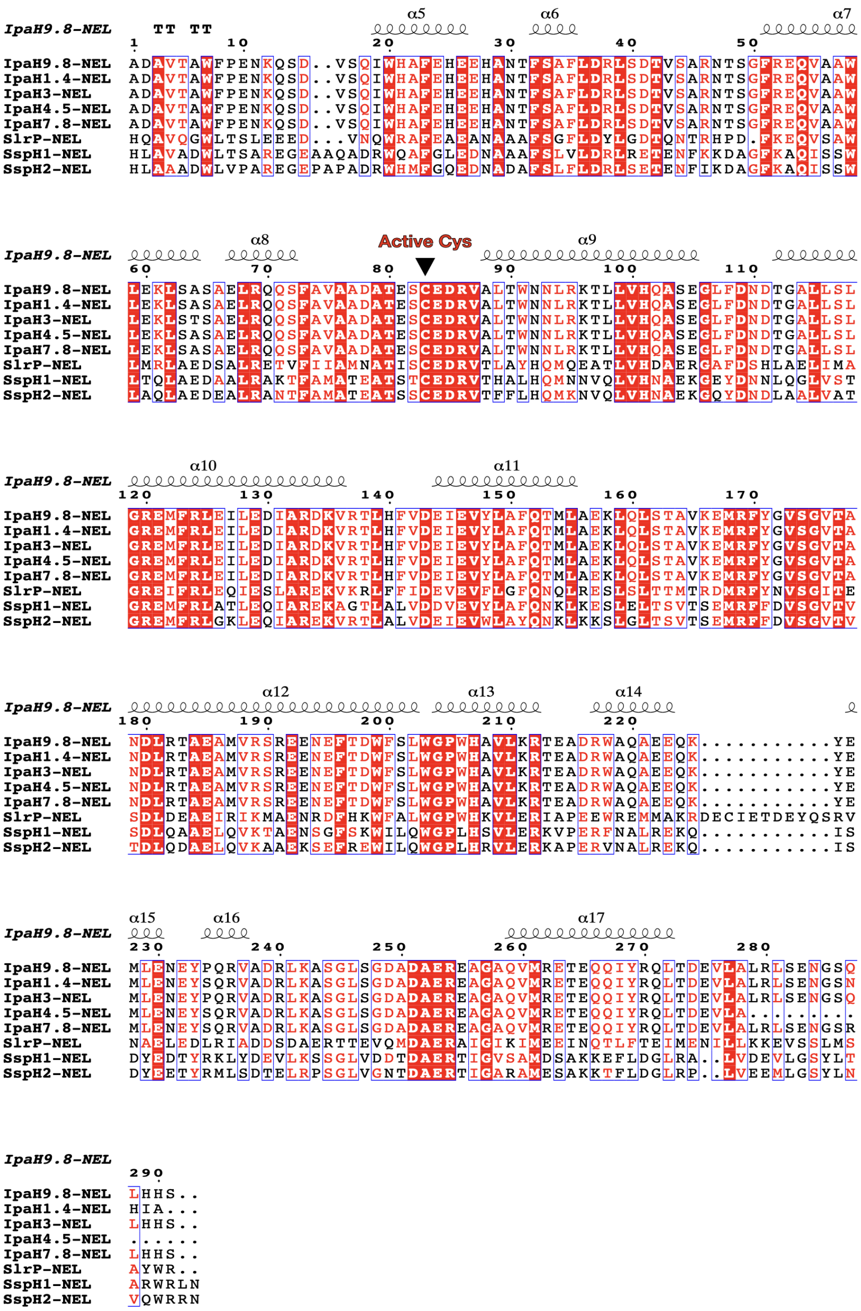

Supplementary Figure 2. ***Sequence alignment of the NEL domains of IpaH enzymes***

Secondary structure elements are labeled and indicated for IpaH9.8 (top). Residues with similar properties are indicated with thin blue transparent boxes and highlighted in red. Strictly conserved residues are shown in red boxes. The conserved active cysteines are indicated by black triangles and labeled as such. The eight enzymes indicated are: IpaH9.8, IpaH1.4, IpaH3, IpaH4.5 and IpaH7.8 from *Shigella flexneri*; SlrP, SspH1 and SspH2 from *Salmonella enterica*.

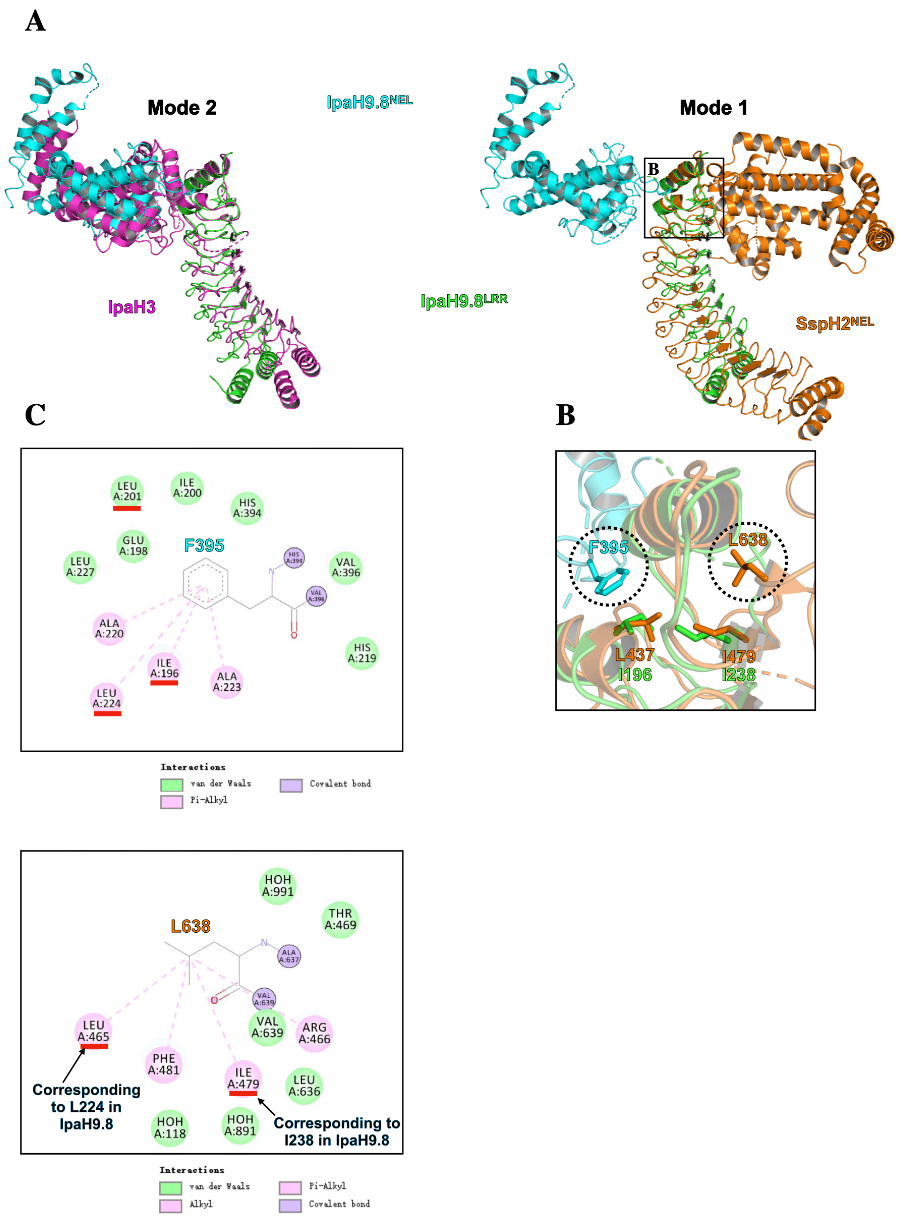

Supplementary Figure 3. ***The LRR-NEL interface in autoinhibition mode 1 and mode 2***

(A). Superposition of the LRR domain of IpaH9.8 (LRR colored green, and NEL colored cyan) to SspH2 (PDBID: 3L3P, colored orange) and IpaH3 (PDBID: 3CVR, colored magenta) revealed that the structure of IpaH9.8 is similar to IpaH3 in mode 2, in which the concave surface is oriented opposite to the NEL domain. (B). The LRR-NEL interface of IpaH9.8 and SspH2. Residues in the LRR and NEL domains of IpaH9.8 are colored green and cyan, respectively. Residues of SspH2 are colored orange. In IpaH9.8, Phe395 interacts with Ile196, which corresponds to Leu437 in SspH2; in SspH2, Leu638 interacts with Ile479, which corresponds to Ile238 in IpaH9.8. (C). *Top*, the 2D visualization diagrams of F395's (top)

interactions with adjacent residues calculated from the IpaH9.8 structure. The residues (Ile196, Leu201 and Leu224) in the hydrophobic cluster are indicated by red lines. *Bottom*, the 2D visualization diagrams of Leu638's (bottom) interactions with adjacent residues calculated from the structure of SspH2 (PDBID: 3L3P). The residues corresponding to the hydrophobic cluster residues in IpaH9.8 were indicated by red lines. Both diagrams were calculated by Molecular Operating Environment. Different types of interactions are indicated under the diagrams. It should be noted that, in IpaH9.8, Ile211 and Leu216 did not interact with Phe395, and that in SspH2, Leu452 (corresponding to Ile211 of IpaH9.8) and Leu457 (corresponding to Leu216 of IpaH9.8) did not interact with Leu638.

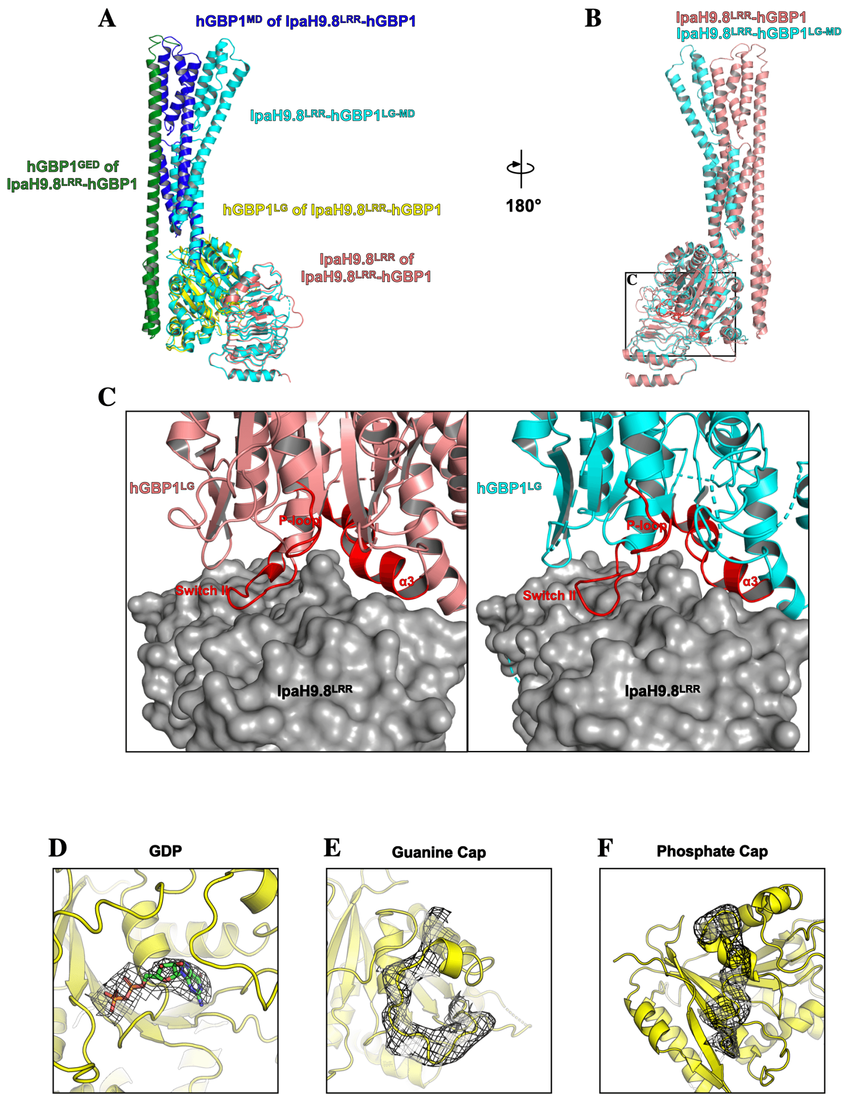

Supplementary Figure 4. ***Comparison of the structures of the IpaH9.8^{LRR}-hGBP1 and IpaH9.8^{LRR}-hGBP1$^{LG\text{-}MD}$ complexes***

(A). Superimposition of the structures of IpaH9.8^{LRR}-hGBP1 and IpaH9.8^{LRR}-hGBP1$^{LG\text{-}MD}$ (PDBID: 6K2D) with IpaH9.8^{LRR}-hGBP1$^{LG\text{-}MD}$ (cyan), with each domain of IpaH9.8^{LRR}-hGBP1 differentiated by colors. (B). Superimposition of the structures of IpaH9.8^{LRR}-hGBP1 and IpaH9.8^{LRR}-hGBP1$^{LG\text{-}MD}$ as in (A) but rotated ~180° with IpaH9.8^{LRR}-hGBP1 in salmon, and IpaH9.8^{LRR}-hGBP1$^{LG\text{-}MD}$ in cyan. (C). Magnified view of the IpaH9.8^{LRR}/hGBP1^{LG} interface with IpaH9.8^{LRR} in gray, and hGBP1^{LG} complexes are differentiated by colors. The P-loop, Switch II, and α3 motifs of hGBP1^{LG} complexes are highlighted in red. (D, E and F) Magnified view of the GDP molecule (D), the guanine cap (E), and the phosphate cap (F) in the structure of IpaH9.8^{LRR}-hGBP1 with 2*Fo-Fc* electron density maps contoured at 1.5 σ. IpaH9.8^{LRR}-hGBP1 is colored yellow. The GDP molecular structure is represented in sticks (C: green, O: red, N: blue, P: orange).

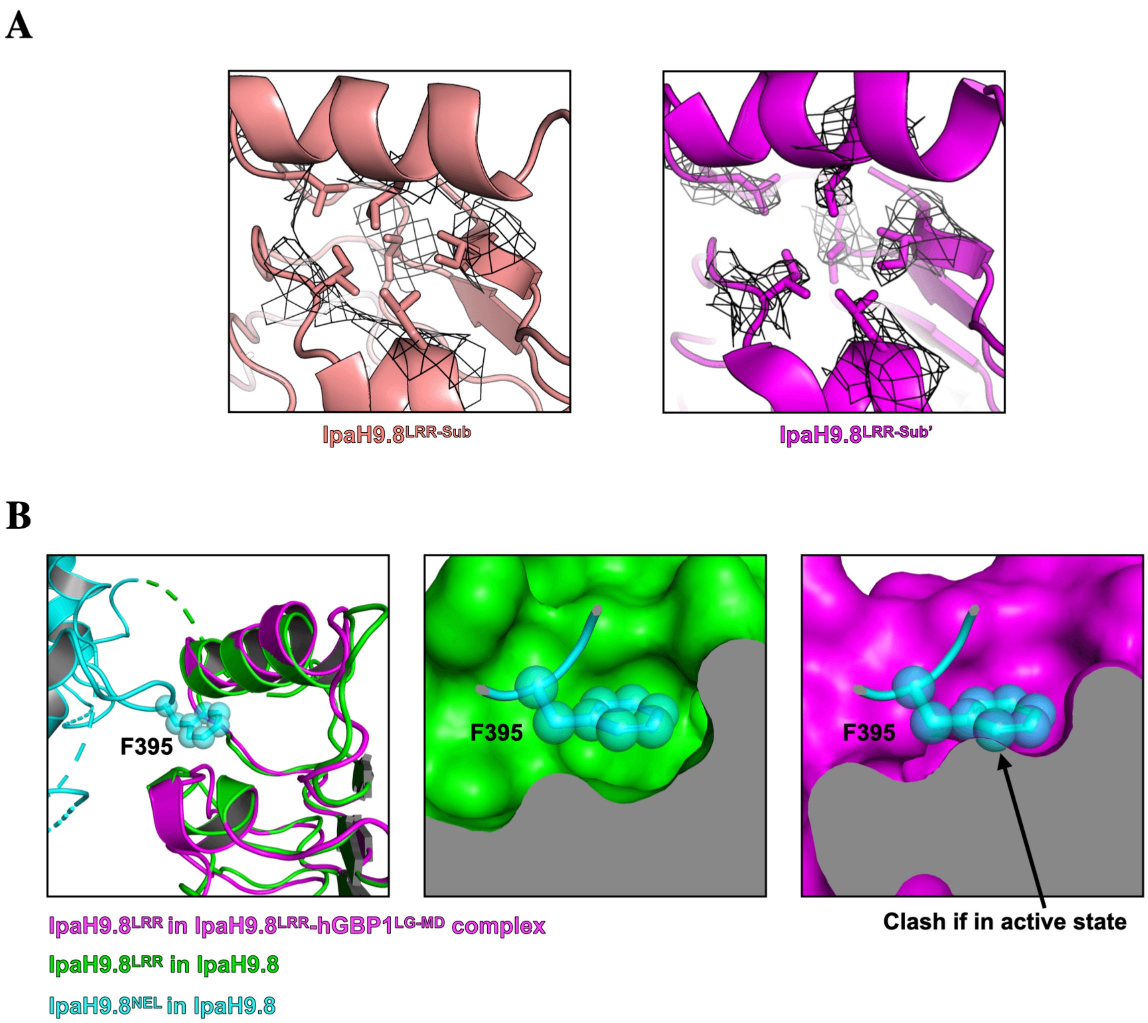

Supplementary Figure 5. ***Comparison of the hydrophobic pockets of LRR-CT in IpaH9.8 and IpaH9.8^{LRR}-hGBP1$^{LG\text{-}MD}$***

(A). Magnified views of the hydrophobic cluster residues in IpaH9.8$^{LRR\text{-}Sub}$ (salmon), IpaH9.8$^{LRR\text{-}Sub'}$ (magenta, PDBID: 6K2D) with 2*Fo-Fc* electron density maps contoured at 1.0 σ. (B). *Left*, magnified view of the LRR-NEL interface of IpaH9.8^{FL} with LRR-CT of IpaH9.8$^{LRR\text{-}Sub'}$ superimposed to IpaH9.8$^{LRR\text{-}FL}$, with each domain colored as indicated. Phe395 of IpaH9.8 ^{FL} was labeled and represented as sticks and spheres. *Middle*, a cross-sectional surface view of the hydrophobic pocket of IpaH9.8$^{LRR\text{-}FL}$ as in *left*. *Right*, a cross-sectional surface view of the hydrophobic pocket of IpaH9.8$^{LRR\text{-}Sub'}$ as in *left*, with the overlapping contact region for IpaH9.8$^{LRR\text{-}Sub'}$ and Phe395 of NEL indicated.

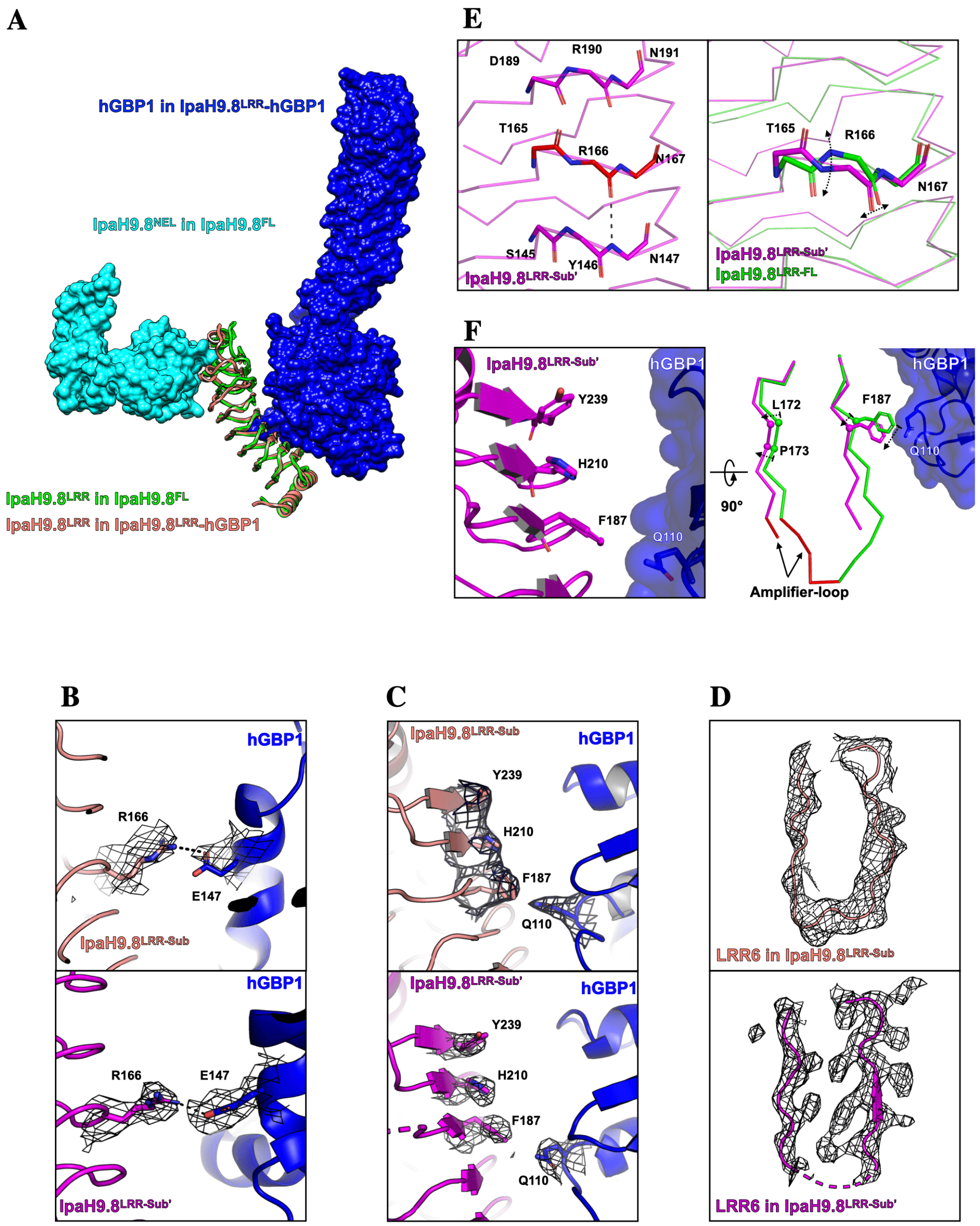

Supplementary Figure 6. ***Arg166 and Phe187 of the LRR domain in IpaH9.8^{LRR}-hGBP1$^{LG\text{-}MD}$ play similar roles as in IpaH9.8^{LRR}-hGBP1***

(A). IpaH9.8^{LRR}-hGBP1 superimposed onto the LRR domain of IpaH9.8^{FL}, with each domain differentiated by color. Two LRR domains are shown as cartoons, while the NEL domain and hGBP1 are shown as surfaces. (B). The interaction between the Glu147 of hGBP1 and the Arg166 of IpaH9.8^{LRR} in IpaH9.8^{LRR}-hGBP1 (*top*) and IpaH9.8^{LRR}-hGBP1$^{LG\text{-}MD}$ (*bottom*) with each protein differentiated by colors and 2*Fo*-*Fc*

electron density maps contoured at 1.0 σ. (C). *Top*, magnified view of LRR in IpaH9.8^{LRR}-hGBP1 that are near Phe187 as in Fig. 6D (*bottom-left*) with 2*Fo-Fc* electron density maps contoured at 1.0 σ. *Bottom*, magnified view of LRR in IpaH9.8^{LRR}-hGBP1$^{\mathrm{LG\text{-}MD}}$ near Phe187 with 2*Fo-Fc* electron density maps contoured at 1.0 σ. (D). The LRR6 motif in IpaH9.8^{LRR}-hGBP1 (*top*) and IpaH9.8^{LRR}-hGBP1$^{\mathrm{LG\text{-}MD}}$ (*bottom*) with 2*Fo-Fc* electron density maps contoured at 1.0 σ. (E) *Left*, magnified view of IpaH9.8$^{\mathrm{LRR\text{-}Sub'}}$ (magenta) near Arg166 as in Fig. 6B (*bottom-left*). *Right*, comparison of the main chain structures of Thr165, Arg166, and Asn167 in IpaH9.8$^{\mathrm{LRR\text{-}Sub'}}$ and IpaH9.8$^{\mathrm{LRR\text{-}FL}}$ as in Fig. 6B (*bottom-right*). (F). Magnified view of IpaH9.8$^{\mathrm{LRR\text{-}Sub'}}$ (salmon, *bottom-left*) near Phe187 as in Fig. 6D (*bottom-left*). *Bottom-right*, comparison of the LRR6 motifs in IpaH9.8$^{\mathrm{LRR\text{-}Sub'}}$ and IpaH9.8$^{\mathrm{LRR\text{-}FL}}$ as in Fig. 6D (*bottom-right*).

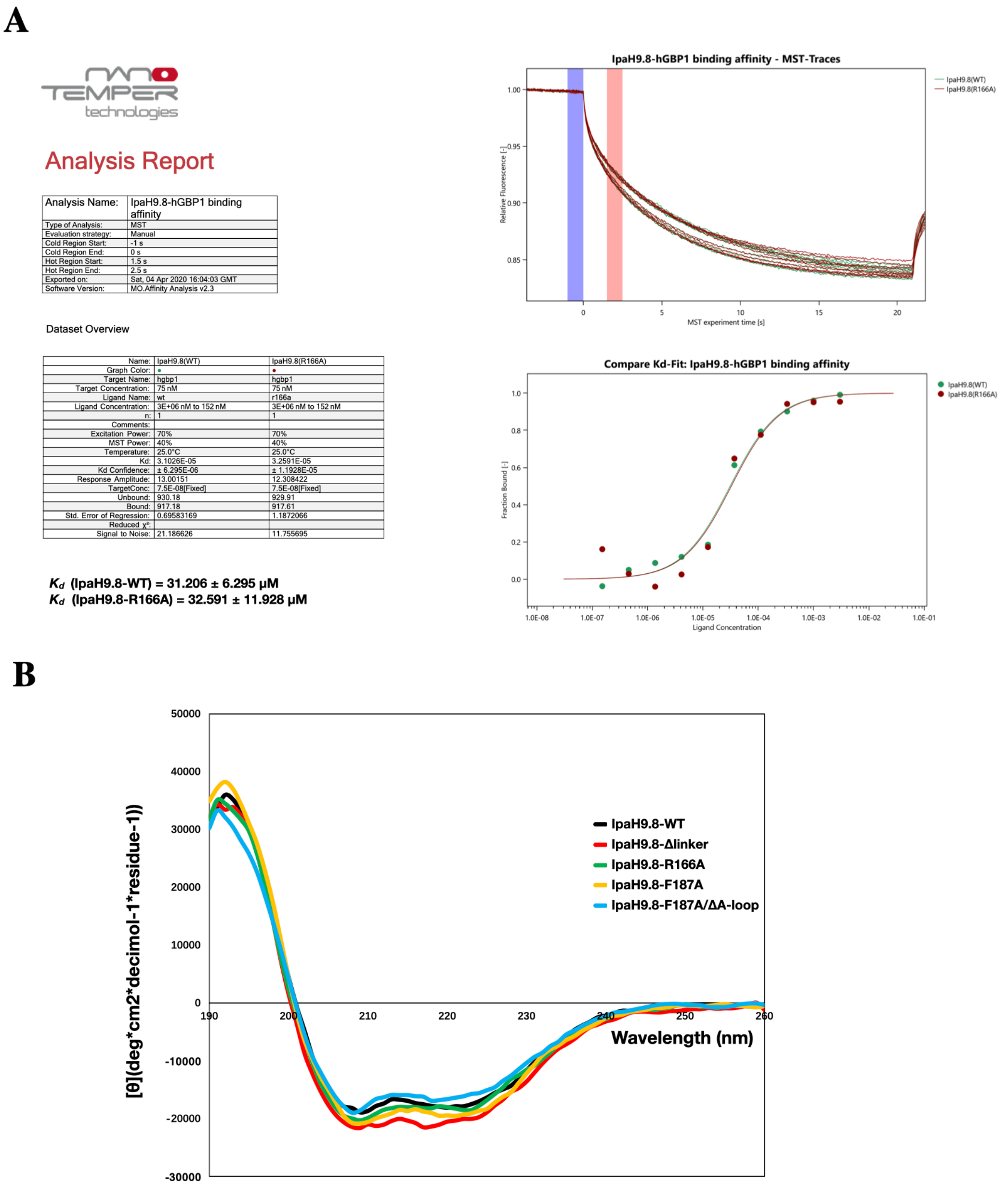

Supplementary Figure 7. ***Microscale thermophoresis (MST) assays and circular dichroism (CD) spectroscopy assays***

(A). Binding analysis of hGBP1 to the indicated wild-type or mutant IpaH9.8 using MST. (B). The CD curves of IpaH9.8, IpaH9.8-Δlinker, IpaH9.8-R166A, IpaH9.8-F187A, and IpaH9.8-F187A/ΔA-loop are colored black, red, green, yellow, and blue, respectively.

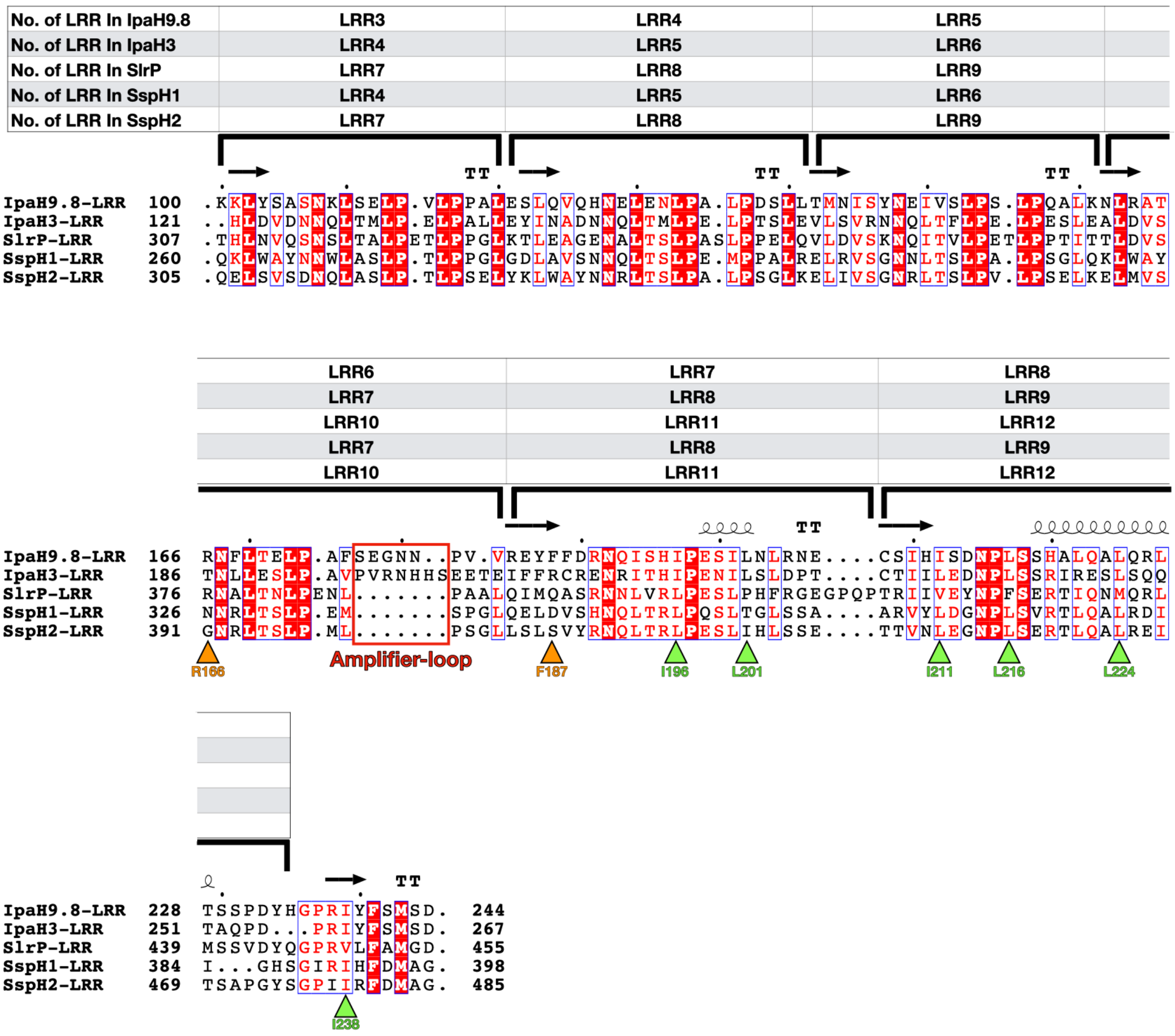

Supplementary Figure 8. ***Structure-based sequence alignment of the LRR domains of IpaH enzymes***

Structure-based sequence alignments of the partial LRR domains of IpaH enzymes. Secondary structure elements are denoted above alignment. The number of LRR motifs for all IpaH enzymes are listed in the table at the top of the alignment. Residues with similar properties are indicated with thin blue transparent boxes and highlighted in red. Strictly conserved residues are shown in red boxes. Arg166 and Phe187 of IpaH9.8 are labeled and indicated by orange triangles. All residues relative to the hydrophobic cluster in IpaH9.8 are labeled and indicated by green triangles. The amplifier-loop region in IpaH9.8 is labeled and indicated by a red box. The five enzymes analyzed were: *Shigella flexneri* IpaH9.8 (in this study), *Shigella flexneri* IpaH3 (PDBID: 3CVR), *Salmonella enterica* SlrP (PDBID: 4PUF), *Salmonella enterica* SspH1 (PDBID: 4NKH), and *Salmonella enterica* SspH2 (PDBID: 3L3P).

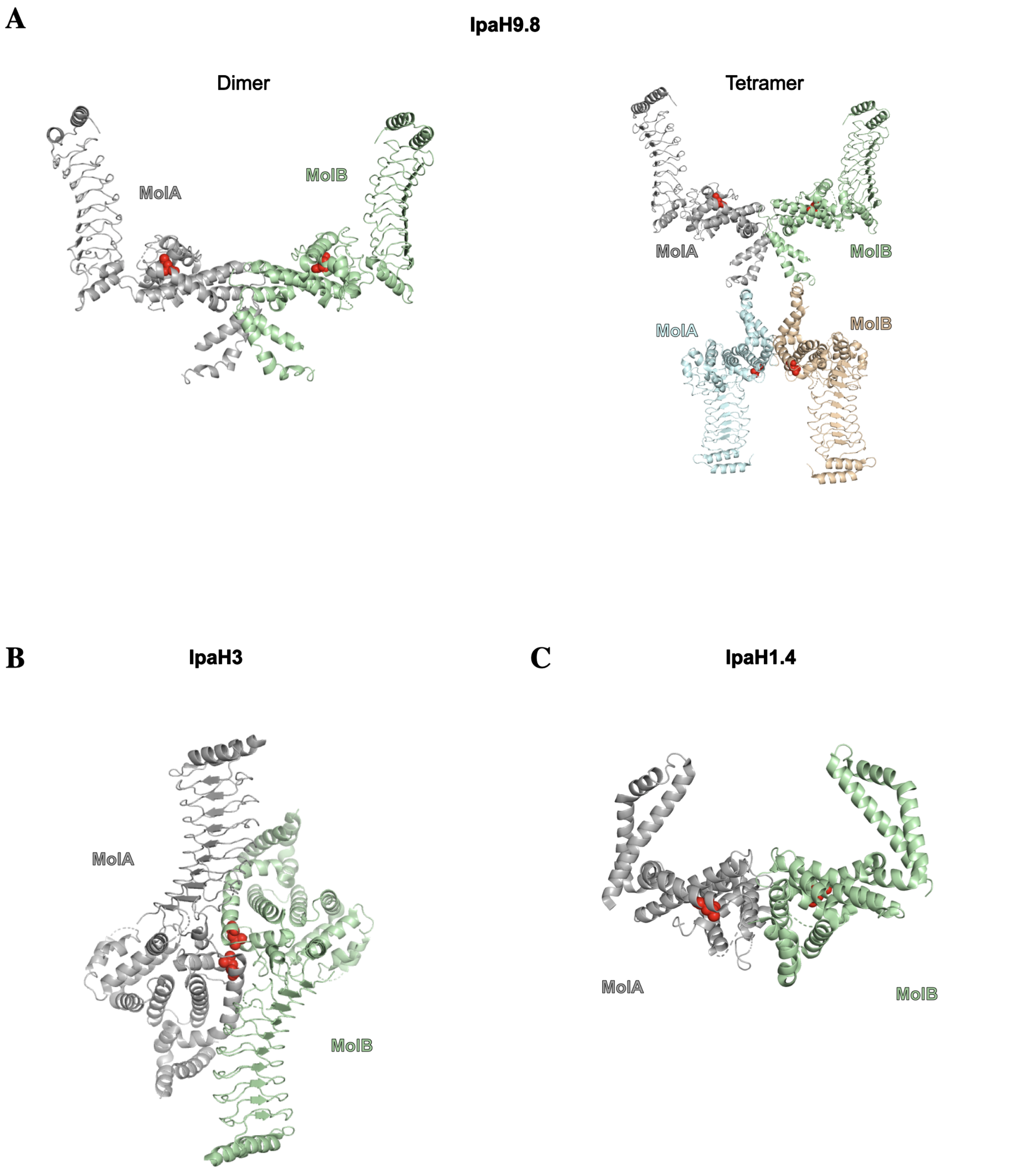

Supplementary Figure 9. ***Various oligomer formation pattern in different IpaH protein crystals***
(A). *Left*, symmetric dimer structure of IpaH9.8 in crystal, with different molecular colored grey and pale-green, respectively. *Right*, symmetric tetramer structure of IpaH9.8 in crystal, with different molecular colored grey, pale green, wheat and pale cyan, respectively. (B). Symmetric dimer structure of IpaH3 in

crystal, with different molecular colored by grey and pale-green, respectively. (C). Symmetric dimer structure of IpaH1.4^{NEL} in crystal, with different molecular colored in grey and pale green, respectively. The Leu461 of IpaH3 in (B), which is inferred to be important to the oligomer formation [46], is shown in red and as spheres. The Leu435 of IpaH9.8 in (A) and the Leu466 of IpaH1.4 in (C), which correspond to the Leu461 of IpaH3, are also shown as red spheres.

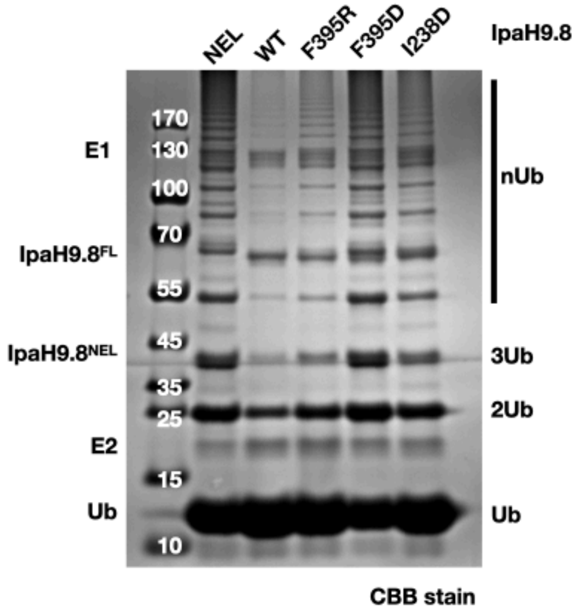

Supplementary Figure 10. ***The mutation of F395D and autoinhibition effect of IpaH9.8***

In vitro ubiquitination activity assays with full-length IpaH9.8 or NEL domain of IpaH9.8. Reaction products were detected using Coomassie brilliant blue (CBB) staining.

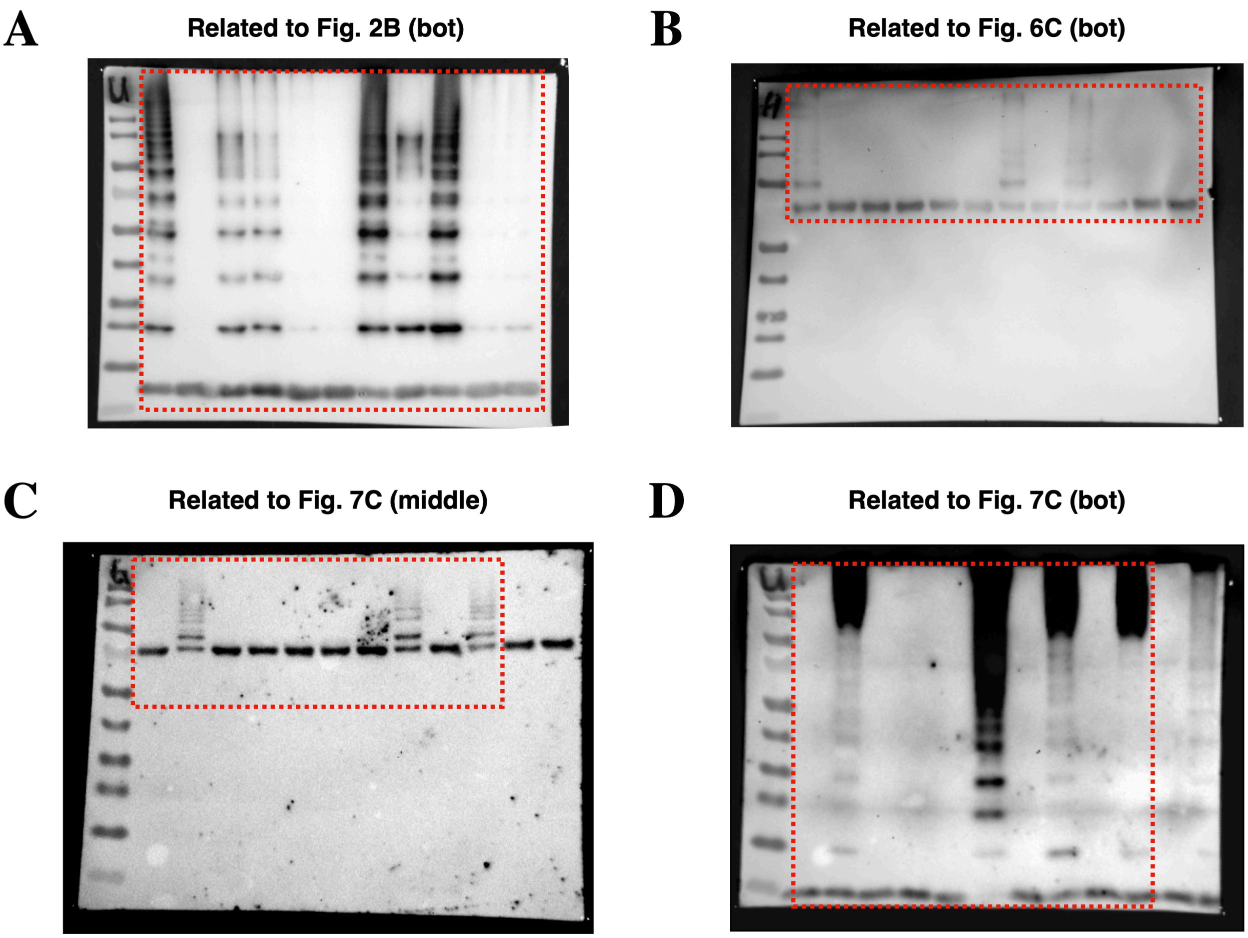

Supplementary Figure 11. ***Western blotting images for Fig. 2B, Fig. 6C, and Fig.7C***

Supplementary Results and Discussion

In the structure of IpaH9.8, Phe395 is surrounded by the hydrophobic cluster of LRR-CT and engages in hydrophobic interactions with the residues in this cluster. Considering the important roles of Phe395, the mutation of F395R should greatly diminish the autoinhibitory effect of IpaH9.8. However, the activity of this mutant is weaker than that of other mutants such as I211D and I238D. We suspect that the mutation of Phe to Arg may not completely disrupt the hydrophobic interactions between Phe395 with the resides in hydrophobic cluster of LRR-CT. In this case, the aliphatic portion of Arg may still engage in hydrophobic interactions with these residues [1,2]. Therefore, we further mutated Phe395 to a more hydrophilic residue Aspartate (F395D). The results show that F395D completely abolished the autoinhibition of IpaH9.8, which showed a more pronounced effect than I238D (**Supplementary Figure 10**). This highlights the importance of the hydrophobic interactions between the hydrophobic cluster of LRR-CT and Phe395 for IpaH9.8 autoinhibition.

Supplementary References

1 Gao, Xiang, et al. Mechanism of substrate recognition and transport by an amino acid antiporter. Nature 463.7282 (2010): 828-832.

2 Petitpas, Isabelle, et al. Crystal structure analysis of warfarin binding to human serum albumin anatomy of drug site I. Journal of Biological Chemistry 276.25 (2001): 22804-22809.
