# Supplementary material for: Substrate-binding destabilizes the hydrophobic cluster to relieve the autoinhibition of bacterial ubiquitin ligase IpaH9.8
Source: Commun Biol. 2020 Dec 10;3:752. doi: 10.1038/s42003-020-01492-1 (PMC7728815; doi:10.1038/s42003-020-01492-1)
Supplement: Supplementary file 3 — Description of Additional Supplementary Files [file 42003_2020_1492_MOESM3_ESM.docx]

Description of Additional Supplementary Files

Supplementary Data 1: Source data underlying plots shown in Figure 6.
